# Synergistic effects of flavonoids and paclitaxel in cancer treatment: a systematic review

**DOI:** 10.1186/s12935-023-03052-z

**Published:** 2023-09-24

**Authors:** Solmaz Asnaashari, Elham Amjad, Babak Sokouti

**Affiliations:** Biotechnology Research Center, Tabriz University of Medical Sciences, Tabriz, Iran

**Keywords:** Paclitaxel, Flavonoid, Anticancer, Synergistic effect, Treatment

## Abstract

Paclitaxel is a natural anticancer compound with minimal toxicity, the capacity to stabilize microtubules, and high efficiency that has remained the standard of treatment alongside platinum-based therapy as a remedy for a variety of different malignancies. In contrast, polyphenols such as flavonoids are also efficient antioxidant and anti-inflammatory and have now been shown to possess potent anticancer properties. Therefore, the synergistic effects of paclitaxel and flavonoids against cancer will be of interest. In this review, we use a Boolean query to comprehensively search the well-known Scopus database for literature research taking the advantage of paclitaxel and flavonoids simultaneously while treating various types of cancer. After retrieving and reviewing the intended investigations based on the input keywords, the anticancer mechanisms of flavonoids and paclitaxel and their synergistic effects on different targets raging from cell lines to animal models are discussed in terms of the corresponding involved signaling transduction. Most studies demonstrated that these signaling pathways will induce apoptotic / pro-apoptotic proteins, which in turn may activate several caspases leading to apoptosis. Finally, it can be concluded that the results of this review may be beneficial in serving as a theoretical foundation and reference for future studies of paclitaxel synthesis, anticancer processes, and clinical applications involving different clinical trials.

## Introduction

Cancer is a common disease responsible for the annual deaths of millions of people around the world, which can progress silently in the body and affect normal life by affecting various organs [1]. While this acute condition has seen significant advances in therapy, there are still several problems to consider. Researchers are making significant efforts to find new therapies to increase the effectiveness of drugs and reduce the side effects of conventional therapies [2]. Despite various cancer treatment methods, including surgery, radiation therapy, endocrine therapy, immunotherapy, and gene therapy chemotherapy remains the most important and common cancer treatment [3–5]. Chemotherapy is associated with a variety of side effects. These complications affect various organs with different intensities, including mild, severe, and life-threatening grades. Immediate adverse effects appear on skin, hair, bone marrow, blood from the gastrointestinal tract, and kidneys, and then spread to essential parts of the body, including the heart, lungs, and brain [6]. A review of previous studies showed that more than 90% of cancer deaths are related to drug resistance. During chemotherapy, multiple drug resistance can occur due to several mechanisms, such as genetic factors, increased drug efflux, increased metabolism of xenobiotics, growth factor, and enhanced DNA repair capacity. These factors can reduce the efficacy of chemotherapy drugs and cause numerous problems in tumor treatment [3, 7].

For thousands of years that humans have widely used natural medicine against various diseases, and today approximately 25% of the main modern medicines come from natural sources [8–11]. Herbal medicines, in particular, have been in the spotlight as a viable alternative to conventional medical care due to the high cost of conventional medicine and the inability of various countries to provide essential medicines. Herbal medicines are also culturally acceptable to people and have good efficacy, safety, low toxicity, and minimal environmental pollution [8, 12–15]. More emphasis should be placed on the fact that integrating herbal medications into conventional medical practices is hampered by the absence of clinical and pharmacological data for most herbal medicinal products [16]. According to sufficient studies on the development of anticancer drugs, compounds of natural origin showed valuable efficacy in tumor prevention and treatment [17, 18]. Currently, there are four widely used plant-based chemotherapeutic drug groups in the global pharmaceutical market, including vinca alkaloids, epipodophyllotoxins, taxane, and camptothecin derivatives [19]. The previous literature has reported some other plant-based structures with significant anticancer properties. For example, flavonoids with several subgroups are the series of polyphenolic components that were introduced as important natural anticancer agents [20, 21] and various molecular mechanisms have been suggested for the anticancer activities of these structures [22].

Paclitaxel or Taxol (C_47_H_51_NO_14_) is a tricyclic diterpenoid structure that belongs to the taxanes chemotherapeutic products of taxanes, which occur naturally in the bark and needles of *Taxus brevifolia* [23]. For advanced ovarian cancer, the FDA allowed the medication in 1992. Paclitaxel has been used to treat breast, colorectal, esophageal, lung, cervical, and prostate cancers as a chemotherapeutic drug since then [23, 24]. It has been classified as anti-neoplastic, antimitotic, and anti-microtubule agents [24].

Despite research and experience, premedication is recommended to prevent hypersensitivity reactions associated with paclitaxel administration. Several hours before injection of paclitaxel, 20 mg of dexamethasone, diphenhydramine, and H2-antagonists are administered orally or intravenously. The only label-based dose schedule recommends taking paclitaxel prophylactically every 3 weeks [25]. The use of dexamethasone to prevent multiple side effects of paclitaxel has been investigated in various studies, and the results of these studies show the effective role of this glucocorticoid medication in the appearance of side effects [26, 27]. However, there is some evidences that the use of dexamethasone in paclitaxel chemotherapy leads to an increase in metastasis [28]. High-throughput drug testing with emerging and clinical oncology combinations of cell lines and patient-derived cells showed that dexamethasone treatment improved sensitivity to various AKT / PI3K-targeted kinase inhibitors, while considerably reducing the efficacy of chemotherapies such as taxanes [29]. Additionally, unfortunately, the use of steroids is associated with various adverse side effects, including severe suppression of the immune system and metabolic changes such as hyperglycemia, which can threaten the survival of patients [30]. Regarding H2-antagonist drugs, there is also evidence that H2-antagonists do not provide any benefit as part of premedication regimens in reducing the incidence of hypersensitivity reactions in paclitaxel-treated patients [31, 32].

According to previous studies combined treatment of paclitaxel with some natural compounds such as curcumin, also reduced adverse effects and increased the chemosensitivity of cancer cells to paclitaxel [33, 34].

Paclitaxel increases tubulin assembly in microtubules and prevents microtubule depolymerization, inhibits cell cycle progression by intervention in the late G2 or M phase, inhibits growth of the mitosis process and cancer cells [23]. Paclitaxel accumulation and efflux through drug transporters, such as P-glycoprotein, are important factors that contribute to drug effectiveness [35]. Common adverse effects of paclitaxel include hair loss, allergic reactions, nausea, vomiting, bone marrow, neutropenia, leukopenia, anemia, arthralgia, myalgia, mucositis, weakness, neuropathy [36]. The severe side effects and drug resistance of paclitaxel have led researchers to an effort to reduce these complications. Various methods have been proposed to reduce these aftereffects, including the use of combination therapy with two or more therapeutic and complementary agents [37, 38]. Consideration of patient nutrition and the use of plant-based diets can have positive effects on the chemotherapy process [39].

The objectives of this research were to (1) analyze the therapeutic benefits of combining paclitaxel with herbal flavonoids and (2) examine the molecular pathways responsible for these outcomes and their processes.

## Synergistic effects of paclitaxel and flavonoids in cancer treatment

In this article, we discuss the findings of a search of the Scopus database for articles using the terms “paclitaxel” and “flavon*” in relation to the treatment of cancer.

**Fig. 1:**
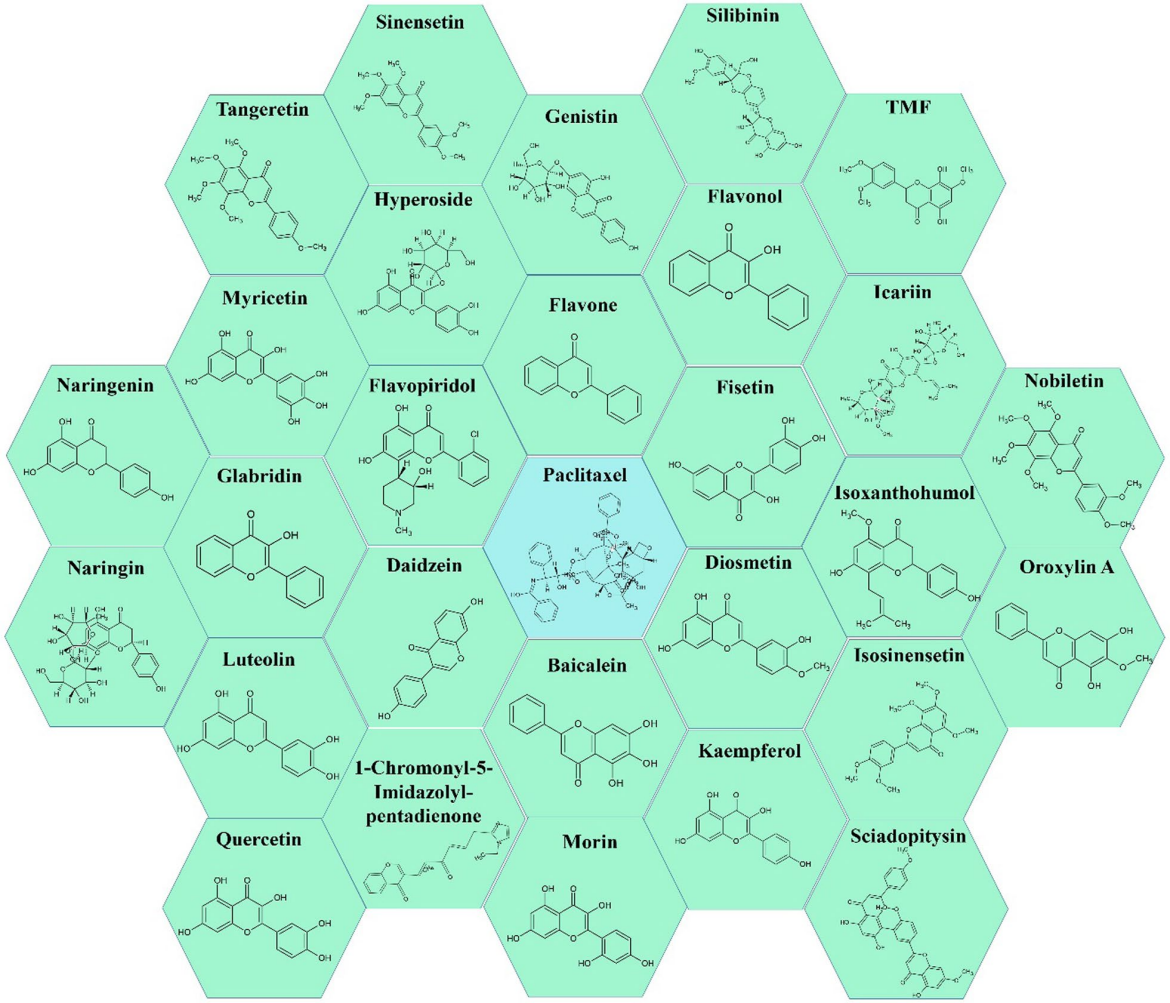
Available flavonoid based compounds synergistically affect paclitaxel in treating various cancers

 Flavonoids are an important group of secondary metabolites of plants with a diphenyl propanoid skeleton (C6-C3-C6) and are classified into several subgroups, such as flavones, flavanones, isoflavones, flavonols, and flavanols (catechins) [40, 41]. Previous studies have shown that flavonoids have several biological and pharmacological effects. Flavonoid structures have several essential effects, one of the most notable being the anticarcinogenic action [40, 41]. Polyphenol chemicals contribute to cell cycle arrest, trigger apoptosis and autophagy, and reduce cancer cell growth and invasion by influencing the activity of enzymes that remove reactive oxygen species [20]. The summarizing of various previous studies on the combination use of flavonoid compounds and paclitaxel as chemotherapy showed the following results (listed in Table 1 and abstracted in Fig. 1):

**Table 1:** Flavonoids and paclitaxel Co-administration results

No.	Flavonoid (cancer type)	Study design	Flavonoid dosage	Paclitaxel Dosage	Duration of study		Mechanism of action	Refs
1	Ampelopsin (Ovarian cancer)	In vitro: A2780, SKOV3, A2780/paclitaxel cells	In vitro: 25, 50, 100 µM	In vitro: 0.01, 0.1, 1µΜ	In vitro: 48 h		Inhibited proliferation Induced G0/G1 and S phase arrest Induced cell apoptosis Activation of p53 Sensitized resistant ovarian cancer cells to paclitaxel through suppression of survivin expression	[44]
2	Apigenin (Cervical cancer	In vitro: HeLa	In vitro: 15 µM	In vitro: 4 nM	In vitro: 24 h		Induction of apoptosis via suppressing the SOD activity led to accumulation of ROS and cleavage of caspase-2	[48]
3	Baicalein (Ovarian cancer)	In vitro: A2780 cells, SKOV3 cells, and OVCAR	In vitro: 1-1000 µM	In vitro: 1-1000 nM	In vitro: 48 h		Anti-tumor effects Increased cell apoptosis and necrosis Increased the caspase-3 activity and its substrate PARP Inhibited cell proliferation through Akt/b-catenin signaling pathway	[52]
4	1-Chromonyl-5-Imidazolylpentadienone (Breast cancer)	In vitro: MDA-MB-231 and MDA-MB-468, T47D	In vitro: 0.5, 1, and 5 µM	In vitro: 1, 5 and 10 nM	In vitro: 24, 48, and 72 h		Induce the anti-proliferative effect and enhance ROS generation in triple-negative breast cancer cells	[54]
	Daidzein (Cervical cancer)	In vitro: A Multi drug resistant cervical carcinoma cell line (KB-V1) and a drug sensitive cervical carcinoma cell line (KB-3-1)	In vitro: 10 and 30 µM	Not clarified	In vitro: 48 h		Increased the multidrug-resistant (KB-V1 with high P-glycoprotein expression sensitivity to vinblastine and paclitaxel in a dose dependent manner and also it could reduce these anti-cancer drugs relative resistance in KB-V1 cell.	[57]
6	Diosmetin (Lung cancer)	In vitro: A549, H1299, H460, SPC-A1, H441, H1650, Calu‐3 In vivo: 4–6 weeks old female BALB/c nude mice (18–20 g;)	In vitro: 5 µM In vivo: 50 mg/kg, three times a week	In vitro: 120 nM In vivo: 10 mg/kg-1, three times a week	In vitro: 48 h In vivo: 4 weeks		Induced ROS-dependent apoptosis via disruption of the PI3K/Akt/ GSK‐3β/Nrf2 pathway and spares normal cells	[60]
	Diosmetin (based on enzyme kinetic, colorectal cancer, and NSCLC)	In vivo: Pooled and mixed human liver microsomes obtained from 25 female and 27 male donors	In vivo: 0.5 to 25 µM	In vivo: 3 to 40 µM	In vivo: 10 min		- Inhibit CYP2C8-mediated paclitaxel metabolism and6-alpha-Hydroxy paclitaxel production.	[61]
7	FD-18 (Breast cancer cells)	In vitro: LCC6 and LCC6MDR In vivo: 4–6 week old athymic nude mice (Balb/c nu/nu), (15–23 g)	In vitro: 1 µM In vivo: 45 mg/kg	In vitro: Not clarified In vivo: 12 mg/kg	In vitro: 5 days In vivo: 12 times in 22 days		Reverses P-gp-mediated multidrug resistance in human breast xenograft in vivo. Increase the accumulation of paclitaxel in LCC6MDR xenograft.	[63]
8	Fisetin (Human liver microsomes)	In vivo: Pooled human liver microsomes	In vivo: 0–25 mM	In vivo: 2.5–25 mM	In vivo: 60 min		Selective reversible and non-competitive inhibitory effect on CYP2C8-mediated paclitaxel hydroxylation	[65]
	Fisetin (Lung cancer)	In vitro: A549	In vitro: 10 µM	In vitro: 0.1 µM	In vitro: 24 h		Reduced the migration and invasion of cancer cells and disruption of the actin and vimentin cytoskeleton structure Inhibition of PI3K/AKT/mTOR signaling pathway	[66]
	Fisetin (Lung cancer)	In vitro: A549	In vitro: 10–50 µM	In vitro: 0.1–0.5 µM	In vitro: 24 h		Reduce the A549 cells viability Prompted low level of apoptosis Cells did not begin the apoptosis cell process despite appearance of G2/M. Activated autophagy	[67]
	Fisetin (Prostate cancer	In vitro: PC-3, DU-145	In vitro: 0–80 µM	In vitro: 10 µM	In vitro: 24, 48, and 72 h		Stabilized microtubules with binding characteristics far superior than paclitaxel. Robust up-regulation of microtubule associated proteins MAP-2 and − 4 α-tubulin acetylation Repressed proliferation, migration, and invasion. Inhibition of Nudc	[68]
9	Flavopiridol (Breast and colon cancer)	In vitro: MCF-7, MDA-MB-468, HCT116 p21	In vitro:150, and 300 nM	In vitro: 100 nM	In vitro: 24 h		Inhibited the spindle inhibitor-induced endoreduplication and polyploidation	[71]
10	Flavone (Human Osteosarcoma)	In vivo: Male Sprague-dawley rats weighing 270–300 g; U2OS and 143B cells	In vivo: 2, 10, 20 mg/kg	In vivo: 40 mg/kg	In vivo: 0, 0.25, 0.5, 1, 2, 3, 4, 8, 12 and 24 h		Enhancement in paclitaxel bioavailability ,inhibition of cytochrome P450 and the p-glycoprotein efflux pump in the intestinal mucosa	[72, 74]
11	Flavanol, 3-hydroxy flavone and dimethoxyderivatives (Human Osteosarcoma)	In vivo: Inbred male Swiss albino mice weighing 20–25 g (U2OS and 143B cells)	In vivo: 25–200 mg/kg	In vivo: A single dose 10 mg/kg	In vivo: 30 min after flavonol administration		Inhibited TNF-α and IL-1β Inhibition of nitric oxide and DPPH radical generation	[74]
12	FV-429 (Lung cancer)	In vitro: human NSCLC cell lines A549 and NCI–H460 In vivo: BALB/c nude mice (18–22 g)	In vitro: Not clarified In vivo: 10 mg/kg	In vitro: Not clarified In vivo: 5 mg/kg	In vitro:24 h In vivo: 2 weeks		Improved the sensitivity of cancerous cells to paclitaxel via the weakening of G2/M phase arrest by deactivating the Wnt pathway Reprogramed hypoxia-inducible factor *1*-alpha-regulated fatty acid metabolism Inhibited the nuclear translocation of β-catenin and blocks cell cycle Enhanced in vivo paclitaxel chemo sensitivity via regulating fatty acid metabolism Yielded better tumor growth suppression	[81]
	FV-429 ( Ovarian cancer)	In vitro: SK-OV-3 and A2780 In vivo: 5-6-week old, female, BALB/c nude mice	In vitro: 5, 10 and 20 µM In vivo: 10 mg/kg	In vitro: 0.2–80 µM In vivo: 5 mg/kg	In vitro: 24 h In vivo: 14 days		Improved the sensitivity to paclitaxel via G2/M arrest promotion. Deteriorated c-Src/Stat3/HIF-1α pathway under hypoxia.	[82]
13	Genistin (Cervical cancer)	In vitro: A Multi drug resistant cervical carcinoma cell line (KB-V1) and a drug sensitive cervical carcinoma cell line (KB-3-1)	In vitro: 10 and 30 µM	Not clarified	In vitro: 48 h		Increased paclitaxel cytotoxicity and decreased the paclitaxel relative Have no modulatory effect on anti-cancer drug cytotoxicity, drug transport or P-glycoprotein expression experiments	[159]
14	Glabridin (Breast cancer cells)	In vitro: MDA-MB-231, MDA-MB-231/MDR1, MCF-7, MCF-7/ADR	In vitro: 10 or 30 µM	In vitro: Not clarified	In vitro: 48 h		Reversing drug that targets P-glycoprotein, which could decrease the IC_50_	[84]
15	Hyperoside (Breast cancer)	In vitro: MDA-MB-231 and HCC1806 cells)	In vitro: 5-100 µg/ml	In vitro: 2–50 nM	In vitro: 24, 48, and 72 h		Improved the effects on apoptosis and caspase-3. Elevate MDA-MB-231 cells sensitivity Muted the TLR4-NF-κB signaling Suppressed apoptosis-related gene and inflammatory cytokine expression Restoring the TLR4 signaling	[93]
16	Icariin (mechanical allodynia through spinal cord as anti-cancer agent)	In vivo: 3- to 4-month-old male Sprague Dawley rats (220 to 250 g)	In vivo: 25–100 mg/kg	In vivo: 8 mg/kg	In vivo: 22 days		Repressed paclitaxel-induced neuro-inflammation and mechanical allodynia in a SIRT1-dependent manner [95].	
17	Isoxanthohumol (Melanoma)	In vitro: B16 and A375 In vivo: syngeneic C57BL/6 mice	In vitro: 0-100 µM In vivo: 20 mg/kg	In vitro: 3.125–25 nM In vivo: 3 mg/kg	In vitro: 2, 6, 12, 24, 48, 72 h In vivo: 10 days		Potent anti-melanoma effects and decreased melanoma cell viability Inhibited melanoma cell division and promoted apoptotic cell death Snsitized melanoma cells to paclitaxel treatment. Targeted the PI3K/Akt and MEK-ERK pathways Inhibited the expression of p70S6K and S6 protein	[99]
18	Isosinensetin (Breast cancer)	In vitro: MX-1 and taxol-resistant MX-1/T cells; MDR1–MDCKII cells for modeling epithelial cells	In vitro: 2 fold of IC50 (IC50: 8.4 µM µM)	In vitro: 75 µM	In vitro: 4 h		Increase taxol cytotoxicity Inhibitory effects on P-glycoprotein	[101]
19	Kaempferol (Cervical cancer)	In vitro: A Multi drug resistant cervical carcinoma cell line (KB-V1) and a drug sensitive cervical carcinoma cell line (KB-3-1)	In vitro: 10 and 30 µM	Not clarified	In vitro: 48 h		Enhanced the multidrug resistance sensitivity with high P-glycoprotein expression Improve the cytotoxic effects and decrease the relative resistance of paclitaxel	[57]
20	Luteolin (Oesophageal cancer)	In vitro: TE-1, EC109, TE-1/PTX, and EC109/PTX cells In vivo: Adult female 4-week-old athymic BALB/c nude mice (15–20 g)	In vitro: 0–40 µM/L In vivo: 50 mg/kg/day	0-256 nM	In vitro: 72 h In vivo: 29 days		Anti-stemness effect was due to reduction of SOX2 expression Inhibition of PI3K/AKT pathway and UBR5-mediated SOX2 protein Inhibitory effect on cell migration by affecting EMT process	[107]
	Luteolin (Oesophageal cancer)	In vitro: TE-1 and EC109 cells In vivo: 4-week-old female BALB/c nude mice (13–14 g)	In vitro: 20 and 30 µM In vivo: 50 mg/kg/day,	In vitro: 2, 5, and 15 nM In vivo: 5 mg/kg/2 day	In vitro: 24 and 48 h In vivo: 19 days		Inhibition of cell migration and EMT processes may be related to the SIRT1 inhibition Induce mitochondrial apoptosis with ROS/JNK pathway	[108]
	Luteolin (Breast cancer)	In vitro: MDA-MB-231	In vitro: 2 µM	10 nM	48 h		Inhibited breast cancer stemness and improves chemosensitivity via Nrf2-Mediated Pathway.	[109]
	Luteolin (Breast cancer)	In vitro: MDA-MB-231 In vivo: 6-week-old female athymic nude mice (BALB/cAnN.Cg-Foxnlnu/CrlNarl)	In vitro:0–15 µM In vivo: 3 mg/kg, 3 times/week	In vitro: 40 nM In vivo: 1 mg/kg,, 3 times/week	In vitro: 24, and 48 h In vivo: 28 days		Activation of caspase-8 and caspase-3 and increasing Fas expression. Blocking of the STAT3 transcription factor	[110]
	Luteolin (Oral squamous cell carcinoma)	In vitro: SCC-4 In vivo: 5-6-week-old male nude mice (BALB/c *nu/nu*) (18–22 g)	In vitro: 0-100 µM In vivo: 5 and 10 mg/kg/2days	In vitro: 0.3 nM In vivo: 1 mg/kg/2 days		In vitro: 24, 48, and 72 h In vivo: 44 days	Decreased the SCC-4 cells viability, induced apoptosis by decreasing the expression of cyclin-dependent kinase (CDKs), cyclins, and phosphor- retinoblastoma (p-Rb) anti-apoptotic protein, echanced the expression of proapoptotic proteins and stimulated caspase 9 and 3, with a concomitant increase in the levels of cleaved poly-ADP-ribose polymerase (PARP)	[105]
21	Morin (Prostate cancer)	In vitro: DU145 and PC-3 In vivo: nude mice	In vitro: 50 µM In vivo: 50 mg/kg	In vitro: 0-100 nM In vivo: 50 µg/kg	In vitro: 48 h In vivo: 20 days		Improve the chemo sensitivity via restoring the miR-155-suppressed expression of GATA3	[113]
22	Myricetin (Ovarian cancer)	In vitro: A2780 and OVCAR3	In vitro: 5 µM	In vitro: 100 nM	In vitro: 48 h		Enhanced the paclitaxel efficacy by targeting multidrug resistance protein-1	[115]
23	Naringenin (Prostate cancer)	In vitro: PC-3 and LNCaP cells	In vitro: 50 µM	In vitro: : 10 µM	In vitro:48 h		Induced apoptosis via regulation of PI3K/AKT and suppression of ERK1/2, P38 and JNK signaling pathways. Induced the MMP loss and ROS generation for intrinsic apoptotic Enhance the paclitaxel efficiency to suppress the cancer cells progression	[117]
24	Naringin (Prostate cancer)	In vitro: DU145, PC3, and LNCaP	In vitro150 mM	In vitro: 5 nM	In vitro: 72 h		Inhibits cell survival and cell migration Induces apoptosis Increases cell cycle arrest Upregulates PTEN and inhibits NF-kB signaling	[120]
25	Nobiletin (Lung cancer)	In vivo: A549/T xenograft model: Male Sprague–Dawley rats (8 weeks old, 180 g), and Balb/c-nude mice (8 weeks old, 20 g)	In vivo: 12.5, 25, 35, and 50 mg/kg	In vivo: 10.5 and 15 mg/kg	In vivo: Every 3 days for 21 days		Reversed paclitaxel resistance in multi-drug resistance Increasing the tumor paclitaxel concentration and modulating Nrf2/AKT/ERK pathways	[124]
26	Oroxylin A (Ovarian cancer)	In vitro: NCI/ADR-RES In vivo: Male Sprague-Dawley rats (280–300 g)	In vitro: 0–40 *µ*M In vivo: 30 mg/kg	In vitro: 5 *µ*M In vivo: 15 mg /kg	In vitro: 72 h In vivo: 0.25, 0.5, 0.75, 1, 2, 4, 8, 12, and 24 h		Inhibitory effect on P-glycoprotein mediated drug efflux	[126]
	Oroxylin A (Breast cancer)	MX-1 and taxol-resistant MX-1/T cells; MDR1–MDCKII cells for modeling epithelial cells	In vitro: 2 fold of IC50 (IC50: 155.6 µM)	In vitro: 75 µM	In vitro: 4 h		Increase taxol cytotoxicity and decrease the cell viability Inhibitory effects on P-glycoprotein	[101]
27	Quercetin (Cervical carcinoma)	In vitro: A Multi drug resistant cervical carcinoma cell line (KB-V1) and a drug sensitive cervical carcinoma cell line (KB-3-1)	In vitro: 10 and 30 µM	Not clarified	In vitro: 48 h		Stimulate the accumulation, and decreased the efflux of Rh123, in KB-V1 cells dose dependently Reduction in Rh123 efflux from cells and resulted in an increase in intracellular Rh123 retention	[57]
	Quercetin (Gastric adenocarcinoma)	In vitro: AGS-cyr61	In vitro: 0-200 µM	In vitro: 0-100 nM	In vitro: 24 h		Reduced multidrug resistance-associated protein 1 and nuclear factor (NF)-kappa B p65 subunit levels Reversed multidrug resistance Reserved colony formation and induced caspase-dependent apoptosis Suppress migration and down-regulated EMT-related proteins in AGS-cyr61	[129]
	Quercetin (Choriocarcinoma Cells)	In vitro: JAR and JEG3	In vitro: 0-100 µM	In vitro: 2.5 and 5 µM	In vitro: 48 h		Inhibition on development of choriocarcinoma cells were mediated via PI3K/AKT and MAPK signal transduction cascades Decreased proliferation and induced cell death, with an enhancement in the cell cycle sub-G1 phase. Induced mitochondrial dysfunction significantly reduced MMP and increased the production of ROS Reserved the phosphorylation of AKT, P70S6K, and S6 proteins, whereas it enhanced phosphorylation of ERK1/2, P38, JNK and P90RSK proteins	[130]
	Quercetin (Basophilic leukemia)	In vitro: RBL-2H3 In vivo: adult male Sprague-Dawley rat (180–220 g) and mice (22–25 g)	In vitro: 3, 10, and 30 µmol/L In vivo: 20 and 60 mg/kg	In vitro: 10 µmol/L In vivo: 2 mg/kg	In vitro: 24 h In vivo: 40 days		Improved the neuropathic pain by stabilizing mast cells and blocking of the PKCε-dependent TRPV1activation	[131]
	Quercetin (Colorectal cancer)	In vitro: HCT116	In vitro:0-100 *µ*M	In vitro: 0–400 nM	In vitro: 24, 48, 72 h		Inhibited the taxol activity to induce G2/M arrest Reduce the cancer cells clonogenicity and survival	[132]
28	Sciadopitysin (Breast cancer)	In vitro: MX-1 and taxol-resistant MX-1/T cells; MDR1–MDCKII cells for modeling epithelial cells	In vitro: : 2 folds of IC50 (IC50: 106.8 µM)	In vitro: : 75 µM	In vitro: 4 h		Increase taxol cytotoxicity and decrease the cell viability Inhibitory effects on P-glycoprotein	[101]
29	Silibinin (Breast cancer)	In vitro: MCF-7	In vitro: 1-400 µM	In vitro: 1-200 nM	In vitro: 24 h		Decreasing in anti-apoptotic Bcl-2 level Increasing in pro-apoptotic Bax, P53, BRCA1 and ATM mRNA levels	[140]
	Silibinin (Renal cancer)	In vitro: 786-O In vivo: 5–6 week-old immuno-deficient nude mice (ICR nu/nu mice) (18–22 g)	In vitro: 0–50 µM In vivo: 100 and 200 mg/kg/day	In vitro:0-200 nM In vivo: -	In vitro: 24, 48 h In vivo:44 days		Decreased MMP-2, MMP-9, u-PA, p-p38, and p-Erk1/2 expressions in a concentration-dependent manner Decreased the NF-kB, c-Jun and c-Fos Enhanced the chemosensitivity of paclitaxel	[141]
30	Sinensetin (Breast cancer)	In vitro: MX-1 and taxol-resistant MX-1/T cells; MDR1–MDCKII cells for modeling epithelial cells	In vitro: : 2 folds of IC50 (IC50: 37.8 µM)	In vitro: : 75 µM	In vitro: 4 h		Increase paclitaxel cytotoxicity Inhibitory effects on P-glycoprotein	[101]
31	Tangeretin (Ovarian and lung cancer)	In vitro: A2780, A2780/T, A549, A549/T	In vitro: 0.83, 2.51, 7.53 µM	In vitro: 1 µM to 0.03 nM, 10 µM to 0.3 nM, or 100 µM to 3 nM	In vitro: 24, 48, 72 h		Increased the chemotherapeutic agents efficacy in ABCB1 overexpressing cells Induced apoptosis Arrested resistant cells at the G2/M-phase Exerted synergistic effect in multidrug resistance cells	[83]
	Tangeretin (Breast cancer)	In vitro: MX-1 and taxol-resistant MX-1/T cells; MDR1–MDCKII cells for modeling epithelial cells	In vitro: 2 folds of IC50 (IC50: 25.3 µM)	In vitro: 75 µM	In vitro: 4 h		Increase paclitaxel cytotoxicity and decrease the cell viability Inhibitory effects on P-glycoprotein	[101]
32	TMF (Colon and lung cancer)	In vitro: Caco-2 and SK-MES-1/PT4000	In vitro: 50–400 µM	In vitro: 0-100 µM	In vitro: 72 h		Improved the bioavailability and enhance paclitaxel cytotoxicity and apical to basolateral transport Apical loading of TMF increased the sensitivity of paclitaxel to overexpressing P-glycoprotein on basolateral side	[147]
33	Vadimezan (Lung cancer)	In vivo: 15 Japanese patients with stage IV advanced non-small cell lung cancer	In vivo: 600–1800 mg/m2	In vivo: paclitaxel (200 mg ⁄ m2) and carboplatin (at a plasma AUC of 6 mg ⁄ ml * min)	In vivo: 6 cycle (Each treatment cycle span was 21 days)		Addition of ASA404 to the standard treatment (paclitaxel and carbopolatin) Decreased adverse effects	[149]
	Vadimezan (Lung cancer)	In vivo: 108 squamous and non-squamous non-small cell lung cancer patients	In vivo: 1200, 1800 mg/m2	paclitaxel (P; 175 mg/m2) and carboplatin (C; AUC 6 mg/ml•min)	In vivo: 6 cycle		Addition of ASA404 to the standard treatment (paclitaxel and carbopolatin) did not increase the toxicity and did not report a serious side effects Addition of ASA404 to the standard treatment could improve the survival rate in both squamous and non-squamous population	[150]

## Ampelopsin (Dihydromyricetin)

Ampelopsin is a natural flavonol structure with six hydroxyl groups. It is the most abundant flavonoid compound of rattan tea (*Ampelopsis grossedentata*) and has protective activities in different organs, including the liver, skin, cardiovascular, and nerve systems [42]. Among several reported biological and pharmacological activities with associated molecular mechanisms of ampelopsin, antitumor effects have received a great deal of attention in in vitro and in vivo studies [42]. Ampelopsin signaling pathways inhibited apoptosis, invasion, migration, proliferation, and cell cycle arrest [43]. Xu et al. found that this natural flavonoid inhibited human ovarian cancer cell multiplication and induced apoptosis. Furthermore, it could noticeably sensitize paclitaxel-resistant ovarian cancer cells to this chemotherapeutic drug by inhibiting the expression of survivin [44]. The results showed that the apoptotic rate increased to 29.25% in the co-treatment of ampelopsin and paclitaxel compared to 17.16% in the treatment with paclitaxel in cells resistant to A2780 / paclitaxel [44].

## Apigenin (4′, 5, 7-trihydroxyflavone)

Apigenin is a naturally occurring flavonoid in common vegetables and fruits with significant antioxidant, anti-inflammatory, chemopreventive and antitumor activities [45]. Several mechanisms have previously been suggested for the anticancer activities of this flavone structure, including induction of apoptosis, autophagy, and immune responses, cell cycle modulation, and cancer cell migration and invasion inhibition. In cancer therapy, apigenin modulated PI3K/AKT/mTOR, MAPK/ERK, NF-κB, JAK/STAT, and Wnt/βcatenin signaling pathways PI3K / AKT / mTOR, MAPK / ERK, NF-κB, JAK / catenin, PI3K / AKT / mTOR, MAPK / ERK, NF-κB, JAK / STAT and Wnt / STAT and Wnt / catenin [46]. For the effectiveness of chemotherapy and based on cancer genetic variation, co-administration of apigenin with other chemotherapeutic drugs was recommended in the previous literature [46, 47]. Xu et al. found that apigenin and paclitaxel had additive effects on apoptosis in cervical cancer cells [48]. Both apigenin and paclitaxel induced cytotoxicity in a dose-dependent manner. 25 µM of apigenin and 10 nM of paclitaxel-induced cytotoxicity with cell viability of approximately 29% and 24%, respectively. The results showed a decrease in cell viability of more than 50% when apigenin (15 moxazie02M) and paclitaxel (4 nM) were administered together [48]. The results suggest that apigenin may reduce SOD function, making HeLa cells more susceptible to paclitaxel-triggered apoptosis. Depolarization of matrix metalloproteinase (MMP) and caspase-2 activation were additional features of apigenin-paclitaxel-induced cancer cell death [48].

Review of other previous studies revealed that synthetic flavonoid homodimers such as apigenin homodimers could increase anticancer drug accumulation, cytotoxicity of chemotherapeutic drugs, and chemosensitivity in resistant cancer cells [49, 50].

## Baicalein (5,6,7-trihydroxy-2-phenyl-4 H-chromen-4-one)

The flavone compound known as baicalein was first isolated from plants belonging to the genus *Scutellaria*. It has three separate hydroxyl groups and a flavone structure. Cardioprotective, anti-inflammatory, and anticancer properties are only a few of the pharmacological benefits of this antioxidant flavonoid [51]. Combined treatment with this flavonoid and paclitaxel exhibited substantially stronger antitumor activities, and prior studies showed that baicalein has considerable antitumor activities in human ovarian cancer cells. Increased cancer cell death can be attributed to the combination of paclitaxel and baicalein through two distinct mechanisms: activation of caspase-3 and cleavage of poly-ADP-ribose polymerase [52]. This study also discussed the nanoparticle form of paclitaxel-baicalein as a different drug delivery method, which could cause synergistic antitumor activities and improve drug resistant problems in human lung cancer cells [52, 53].

## 1-Chromonyl-5-Imidazolylpentadienone

1-Chromonyl-5-Imidazolylpentadienone or 3-((1*E*, 4*E*-5-(1-ethyl-1* H*-imidazol-2-yl)-3-oxopenta-1,4-dien-1-yl)-4* H*-chromen-4-one introduced as a synthetic hybrid structure, which was obtained by integrating several advantaged pharmacophores (e.g., curcumin and quercetin) into a single compound [54]. In MDA-MB-231 and MDA-MB-468 cell lines related to triple negative breast cancer cells, this compound has the potential to inhibit cell development, improve mitochondrial reactive oxygen species, and decrease the level of EMT level by modification of E-cadherin and N-cadherin as EMT indicators [54]. 1-Chromonyl-5-Imidazolylpentadienone showed synergism with Paclitaxel and anticancer effectiveness against triple negative breast cancer cells [54].

## Daidzein (4′, 7-dihydroxyisoflavone)

Daidzein is known as a natural phytoestrogen compound with a non-steroidal chemical structure and is derived mainly from soybeans and mung bean. This polyphenol is similar to mammalian estrogens in terms of chemical structure and can replace or interfere with estrogens and their receptor complex. It shows protective activities against several diseases related to estrogen regulation including breast cancer, cardiovascular disease, osteoporosis, diseases, and diabetes disorders [55]. Daidzein can affect some independent biological activities and showed other different beneficial effects such as anticancer, antioxidant, anti-inflammatory, skin protective, and neuroprotective effects [56]. Using daidzein increased the sensitivity of vinblastine and paclitaxel-resistant human cervical cancer cells (KB-V1) with high expression of P-glycoproteins, as reported in a research published in 2005. This may also reduce drug resistance in KB-V1 cells, which would be beneficial in the treatment of cancer [57].

A recently published study evaluated the pharmacological characteristics of daidzein in reducing paclitaxel-induced neuropathic pain, and the results obtained demonstrated that daidzein administration could downregulate the TRPV1 and P2Y, therefore reduced hyperalgesia. Furthermore, this isoflavone structure increased Nrf2 (nuclear factor erythroid-2-related factor 2) and HO-1 (heme oxygenase-1) proteins, and played a vital part in the activation of antioxidant pathway. It could also decrease neuronal apoptosis through the reduction of caspase-3 and BAX (Bcl2-associated X-protein), while increasing Bcl-2, simultaneously. Daidezein moderated the severe DNA damage caused by paclitaxel. Furthermore, inhibited neuroinflammation by increasing the anti-oxidant enzymes and decreasing oxidative stress markers. Daidzein also suppressed pro-inflammatory mediators. in conclusion, daidzein showed substantial neuroprotective effects against paclitaxel- induced neuropathic pain [58].

## Diosmetin (3′,5,7-trihydroxy-4′-methoxyflavone)

Diosmetin is known as a methoxyflavonoid structure that is isolated from the citrus genus. Antimicrobial, anti-diabetic, anti-inflammatory, antioxidant, and antitumorigenesis properties were some examples of the wide-ranging therapeutic effects of this bioflavonoid [59]. When lung cancer cells received diosmetin and paclitaxel, the effects were antiproliferative and cytotoxic. Diosmetin could induce selective apoptosis and improve the paclitaxel chemotherapeutic ability of NSCLC cells through ROS accumulation of ROS through the PI3K/Akt/GSK-3β/Nrf2 pathway [60]. According to molecular coupling simulations and enzyme kinetic studies, diosmetin inhibited CYP2C8-mediated paclitaxel metabolism and could inhibit the production of 6-alpha-hydroxy paclitaxel as the main inactive metabolite of paclitaxel [61].

## FD-18

amine-linked flavonoid dimers, FD-18, are introduced as a new, potent, and safe synthetic flavonoid structure with P-glycoprotein modulating activity that can prevent drug resistance in cancer [62]. Previous reports showed that FD-18 at a concentration of 140 nM could reverse the resistance of paclitaxel [62]. Yan et al. presented the flavonoid dimer as a strong P-glycoprotein modulator for the clinical management of P-glycoprotein-mediated multidrug resistance cancers [63]. They showed that co-administration of FD-18 and paclitaxel caused a 46% decrease in LCC6MDR xenograft volume. The results indicated that FD18 could increase the accumulation of paclitaxel in the LCC6MDR xenograft [63].

##  Fisetin (3,7,3′,4′-tetrahydroxyflavone)

The pharmacological actions of fisetin, a polyphenol present in many types of fruits and vegetables, are many. Antioxidant, anti-inflammatory, and cancer-fighting potentials were reported as these bioflavonoid properties [64]. Possible mechanisms of action include suppression of the PKC/ROS/ERK1/2 and p38 MAPK signaling pathways, attenuation of NF-κB activation, and decrease in oncoprotein securing levels, as well as slowing of cell cycle progression and cell proliferation [64]. In 2018, scientists discovered that the 8 subfamily C member of the cytochrome P450 family 2 (CYP2C8) handled paclitaxel hydroxylation and that fisetin and its methylated metabolite, geraldol, could selectively block CYP2C8 activity in human liver microsomes (the subestrate of the CYP2C8 protein) [65]. Another in vitro study found that when combined with clinically feasible doses of paclitaxel, fisetin reduced proliferation and accelerated cell death in A549 non-small cell lung cancer cells (NSCLC) [66]. By affecting the expression of genes involved in metastasis and changing the structure of the actin and vimentin cytoskeletons, fisetin and paclitaxel together reduced cancer cell motility and invasion. Compared to only fisetin and paclitaxel, the outcomes shown here were much better. The toxic effects of paclitaxel alone on normal cells were greater than the combination of two agents, which showed that fisetin could provide protection against paclitaxel-mediated cytotoxicity [66]. Another research with comparable findings found that these two chemicals had additive effects on A549 NSCLC cells. The findings revealed that the mechanisms of reported synergistic effects include mitotic catastrophe induction through the promotion and formation of multipolar spindles, the elimination of cells with mitotic catastrophe by autophagy, and a noticeable improvement in the level of autophagy [67]. When A549 cells were treated with fisetin or paclitaxel alone, protective autophagy was activated; however, when both drugs were used together, cancer cell autophagy changed to one resulting in their death [67]. Whether used alone or in combination with other chemotherapeutics, fisetin has been proposed by Mukhtar et al. as a potential therapy for prostate cancer [68]. With the introduction of fisetin, a microtubule stabilizing drug with binding qualities other than paclitaxel, microtubules were stabilized by bonding to tubulin. Fisetin induced an upregulation of MAP-2 and MAP-4, two proteins involved in microtubule organization, in prostate cancer cells. Additionally, acetylation of -tubulin was increased in cells treated with fisetin, suggesting microtubule stability [68]. Fisetin therapy reduced the growth, invasion, and metastasis of prostate cancer cells. The protein Nudc, which is part of the dynein/dynactin motor complex, may be inhibited by this. Nudc controls how microtubules move. When tested in the NCI/ADR-RES cell line, fisetin also suppressed cell viability and colony formation [68].

## Flavopiridol

Flavopiridol or alvocidib is a synthetic flavonoid alkaloid obtained from some species of the Meliaceae and Rubiaceae families. It was introduced as an important beneficial agent in combination therapy for the treatment of chronic lymphocytic and acute myeloid leukemia [69]. This compound acted as a strong cyclin-dependent kinase (CDK) inhibitor and also could affect EGFR, pp60 Src, PKC, and Erk-1 [70]. Previous studies have shown synergistic effects between flavopiridol and paclitaxel in NSCLC cells. In addition, flavopiridol observed sequentially dependent caspase activation and apoptosis in a sequentially dependent manner in paclitaxel-treated breast and gastric cancer cells [71]. Chromosomal abnormalities, aneuploidy, and genomic instability have resulted from defects at cell cycle checkpoints, which have been crucial to tumor development. Motwani et al. found that microtubule inhibitors (such as paclitaxel) caused cells with a broken G1 checkpoint to endoreplicate and become polyploid. Polyploid cells could change and lose chromosomes at random to become aneuploid. Flavopiridol stopped cancer cells from endo-reduplication and polyploidization caused by spindle inhibitors [71]. Therefore, this synthetic flavone structure protected the stability of the genome by stopping endo-reduplication and polyploidy. It also showed that it could be used as a chemopreventive drug to stop neoplasia from happening [71].

## Flavone

Flavone or 2-phenyl-4 H-1-benzopyran-4-one is one of the simplest classes of flavonoid and was reported in several types of cereals and vegetables (e.g., dill). Various biological activities from the flavone structure, including the promotion of apoptosis, antiproliferative and antitumor activities [72]. Oral coadministration of flavone and paclitaxel in rats was shown to increase paclitaxel bioavailability. This increase in bioavailability could be attributed to suppression of cytochrome P450 and the efflux pump in the intestinal mucosa [72]. A 2006 published study showed that flavone could inhibit the transport of P-glycoprotein-mediated taxol. [73].

## Flavonol (3-hydroxy flavone)11. Flavonol (3-hydroxy flavone)

3-hydroxy flavone is the simplest structure of the main class of flavonoid family called flavonols, which is obtained synthetically and does not exist in this simple form in nature [74]. The anticancer and antimetastatic effects of 3-hydroxy flavone were described in previous studies [74, 75]. Additionally, EMT, MMP-2, FAK, Src, MEK/ERK, MLC-2 were the pathways involved and reported of 3-hydroxy flavone [74]. Using an animal model of paclitaxel-induced peripheral neuropathy, Sayeli et al. demonstrated that flavonol (3-hydroxy flavone) and its dimethoxy derivatives (3’,4’-dimethoxy flavone, 7,2’-dimethoxy flavone, 6,3’-dimethoxy flavone and 7,3’-dimethoxy flavone) significantly improved signs. Inflammatory cytokines such as IL-1 and IL-6 are stifled by researchers, theorizing that free radicals played a role in the excellent results of the study by stifling the production of pro-inflammatory cytokines (such as TNF-α and IL-1) [76].

## FV-429

FV-429 is a synthetic flavonoid structure and a derivative of woganin (with an O-methylated flavone skeleton, isolated from *Scutellaria baicalensis*) [77, 78]. There is evidence to suggest that FV-429, by causing dysregulation of lysosomes, may prevent autophagy and lysosome-dependent cell death in T cell malignancies [78]. It could sensitize cancer cells to chemotherapy drugs and was suggested as a novel compound with a potent inhibitory autophagy potential and remarkable antitumor ability [78]. Zhou et al. showed in two separate investigations that ROS may induce apoptosis by nuclear translocation of ERK2 and activation of p53 in gastric cancer cells, and that suppression of hexokinase II Akt phosphorylation can induce apoptosis and block glycolysis in breast cancer cells [79, 80]. A recent study has shown that FV-429 may reduce the phase arrest of G2 / M in NSCLC cells by deactivating the Wnt pathway, making cancer cells more sensitive to the drug [81]. In vivo investigations showed that paclitaxel plus FV-429 dramatically suppressed tumor growth in mice with NCI-H460 and A549 tumors [81]. By inhibiting expression and activation, blocking nuclear translocation and HIF-1 binding, and increasing arrest of the G2 / M cell cycle in hypoxic microenvironment-induced resistance of human epithelial ovarian cancer cells to paclitaxel, FV-429 could reverse hypoxic microenvironment-induced resistance to paclitaxel [82].

## Genistin (Genistein 7-glucoside)

Biologically active isoflavone glycoside genistin is extracted from soybeans and kudzu. Some of the many health benefits of genistin are that it lowers the risk of osteoporosis and eases the symptoms of menopause. It also has antioxidant, cardioprotective, hepatoprotective, neuroprotective, antimicrobial, antiapoptotic and anticancer properties [57]. Based on what has been learned from previous studies, controlling the PI3K/Akt/mTOR pathways can affect how well cancer cells can move and spread to nearby tissues. It may inhibit cancer cell proliferation by inducing apoptosis and stopping the cell cycle at the G1 or G2/M checkpoints [57]. Limtrakul et al. showed that genistin could increase paclitaxel cytotoxicity and decrease paclitaxel relative resistance in multidrug-resistant human cervical cancer cells (KB-1) at a concentration of 30 nM. Additionally, anticancer drug cytotoxicity, drug transport, and P-glycoprotein expression were tested in cervical carcinoma KB-V1 and KB-3-1 cells, both of which are drug-sensitive cell lines. Neither of these cell lines showed any signs of modulatory impact of the compound [57].

## Glabridin

The roots of Glycyrrhiza glabra are where you can get the prenylated isoflavone known as glabridin. This isoflavonoid molecule has been linked to a variety of beneficial biological effects, including anti-inflammatory, antioxidant, neuroprotective, estrogenic, anti-osteoporotic, control of energy expenditure and metabolism, chemopreventive, and anticancer effects [83]. Based on an in vitro study in 2019, glabridin could reduce paclitaxel and doxorubicin IC_50_ in breast cancer cells. This study clearly exposed that glabridin could act as an agent that resensitized overexpression of P-glycoproteins to chemotherapeutic drugs in multidrug resistant cancer cells [84].

## Hyperoside (quercetin-3- ***O*-β-*D*-galactopyranoside)**

Hyperoside is a structure of flavonol glycosides found in different plant genera such as *Hypericum* and *Crataegus*. This compound displayed a wide range of biological and pharmacological effects, including antioxidant, anti-inflammatory, anticancer, neuroprotective, and hepatoprotective properties, by targeting multiple molecular pathways, such as NF-κB, PHLPP2, Nrf2-ARE, MAPK, AKT, TGF-β, and nitric oxide signaling. [85–92]. According to the study by Sun et al., concomitant administration of hyperoside and paclitaxel showed protection against paclitaxel-induced cytotoxic effects in mammary gland epithelial cell lines (MCF-10 A). In breast cancer cells (MDA-MB-231 positive for TLR4), the presence of hyperoside increased apoptosis, decreased cell viability, and activated caspase-3, all of which made cancer cells more susceptible to the chemotherapy drug paclitaxel. However, hyperoside administration could not induce the significant sensitivity of paclitaxel in TLR4-null HCC1806. This flavonol reversed the paclitaxel-activated TLR4-NF-κB signaling, weakened the expression of the paclitaxel-interceded anti-apoptotic Bcl2 gene. However, it may enhance the effects on the MDA-MB-231 cell line’s expression of the pro-apoptotic gene bax in the MDA-MB-231 cell line and its levels of the pro-inflammatory cytokine IL6 [93]. The results showed that hyperoside may increase the sensitivity of cancer cells to paclitaxel by blocking pro-inflammatory and pro-survival strategies mediated by TLR4 activation mediated by TLR4, validating the beneficial combination to achieve a suitable chemosensitivity in breast cancer [93].

## Icariin (8-prenyl derivative of kaempferol 3,7- ***O*** -diglucoside)

Icariin is a prenylated flavonol glycoside and a biologically active constituent found in *Epimedii* species. Neuroprotective, cardioprotective, anti-osteoporosis, anti-inflammatory, reproductive system improvement, antioxidant, antidepressant, and antitumor activities were reported as multiple pharmacological properties of this flavonol structure [94]. Paclitaxel administration resulted in a discernible decrease in mechanical thresholds, activation of NF-kB p65, and elevation of TNF-α, IL-1b, and IL-6 cytokines, and activation of astrocytes in the spinal cord. Administration of icariin could alleviate paclitaxel-induced mechanical allodynia and spinal neuroinflammation. This flavonoid reversed the down-regulation of paclitaxel-induced SIRT1 (Spinal Sirtuin 1) and H4 (histone 4) acetylation. Therefore, icariin reduced paclitaxel-induced neuropathic pain as one of the common adverse effects [95].

Several cell lines also produced synergistic effects when icariside II was combined with drugs such as bortezomib, thalidomide and paclitaxel. Icariside II showed inhibition of cancer cell apoptosis by inhibition of STAT3 and TLR4-MyD88-ERK signaling in response to these chemotherapeutic agents [96, 97].

## Isoxanthohumol (Sophora)

Isoxanthohumol is a considerable prenylflavonoid that was found in hops (*Humulus lupulus*) along with two other compounds, xanthohumol and 8-prenylnaringenin [98]. Prenylflavonoids are introduced as the third largest group of phytostrogens, after the isoflavone and lignan structures. *The roots of Sophora flavescens* are the other main natural origin of isoxanthohumol. This polyphenol structure did not show any strong estrogenic activities, but it has been reported to be an apoptosis activator, an antiproliferative, antiangiogenesis, and anticarcinogenic agent [98, 99]. Furthermore, isoxanthohumol was reported to reduce TGF-β expression in MDA-MB-231 breast cancer cells and could interfere with the monoblastic leukemia cell line through the JAK / STAT pathway and inhibit pro-inflammatory gene expression. Antiviral effects against herpes and bovine viral diarrhea viruses have also reported as the biological activities of isoxanthohumol [98]. Based on a 2016 published report, this prenylflavonoid structure strongly sensitized melanoma cells to paclitaxel treatment. Isoxanthohumol showed significant anti-melanoma activity through differentiation induction along with apoptotic cell death. Treatment of mouse B16 cell lines with isoxanthohumol displayed a melanocytic profile and an improved tyrosinase effect without increasing the melanin content. With the A375 melanoma cell line, isoxanthohumol could suppress the activity of β-catenin, Notch 1, and Oct-3/4. This flavonoid compound targeted the PI3K/Akt and MEK-ERK pathways [99].

## Isosinensetin (6-Demethoxynobiletin)

As a polymethoxylated flavone structure, isosinensetin can be found in a wide variety of plant species, including orange, and has been linked to a variety of health benefits [100]. P-glycoprotein in MDR1-MDCKII cells was significantly inhibited by isosinensetin, resulting in a reduction in the P-glycoprotein-mediated efflux of taxol and an increase in cellular toxicity. Taxol cytotoxicity in MX-1 and MX-1/T cells may be further enhanced by isosinensetin [101].

Various studies on nobiltin and its derivatives show that these compounds have the ability to increase the anticancer effects of paclitaxel. Most of these compounds had improved sensitivity to paclitaxel in multidrug resistance P-glycoprotein cancer cells. Furthermore, previous evaluations confirmed that activated NRF2/PI3K/AKT pathways in multidrug resistant cancer cells were incredibly inhibited by nobiletin derivatives and paclitaxel [102]. Similarly, a modest dose of paclitaxel and 5-demethylnobiletin was shown to have synergistic anticancer effects in CL1-5 lung cancer cells, with the results showing a simultaneous reduction in cell survival and increased apoptosis. It was observed in cancer cells. This study suggested that 5-demethylnobiletin cooperated with paclitaxel to induce apoptosis through the caspase pathway by regulating caspase-3, caspase-8, and caspase-9 actions. In addition to this, research on animals showed that a therapy consisting of 5-demethylnobiletin and paclitaxel was capable of significantly suppressing tumor development [103]. Overall, the results suggested that the synergistic effects of the structures of paclitaxel and polymethoxylated flavone in cancer could be suitable topics for particular attention and designated the opportunity to developing supplementary new strategies for control and treatment of different types of cancer.

##  Kaempferol (3,4′,5,7-tetrahydroxyflavone)

Kaempferol is introduced as one of the common naturally occurring flavonol structures, and its chemopreventive and anticancer potency and anti-inflammatory, cardioprotective, and neuroprotective effects have been reported in the previous literature [104]. Kaempferol obviously enhanced the multidrug resistance sensitivity of cervical cancer cells with high expression of paclitaxel and vinbelastine in a dose-dependent manner. It could considerably improve the cytotoxic effects and decrease the relative resistance of paclitaxel [57]. Furthermore, kaempferol could stimulate the accumulation and decrease the efflux of rhodamine-123 123 in KB-V1 cervical cancer cells KB-V1. This study explained that the absence of the hydroxyl group in the 3 ‘position of the B ring in the molecular structure significantly increased the inhibition properties of P-glycoproteins in the function and expression of multidrug-resistant KB-V1 cervical cancer cells [57].

## Luteolin (3’, 4’, 5, 7-tetrahydroxyflavone)

The flavonoid derivative luteolin is found in vegetables, fruits, and herbal remedies, and has a wide range of biological and pharmacological benefits, such as anti-inflammatory, antioxidant, and anticancer properties [105, 106]. It can fundamentally inhibit tumor progression through the intervention of several essential signals and transcription pathways of cancer cells.

According to Zhao et al., luteolin was introduced as a suitable agent for paclitaxel-resistant oesophageal cancer therapy. They reported that luteolin significantly reduced stem-like properties of paclitaxel resistant cancer cells by downregulating SOX2 protein expression and could prevent the PI3K/AKT pathway to reduce AKT (S473) and UBR5 expression, which could promote SOX2 degradation [107]. Due to its flavonoid structure, this compound has the potential to halt the EMT (epithelial-mesenchymal transition) process, thus preventing the migration and invasion of paclitaxel-resistant cancer cells. The tumorigenicity of paclitacel-resistant oesophageal cancer cells was also inhibited by luteolin and this effect was achieved without significant in vivo damage [107]. Furthermore, as Qin et al. study, luteolin could have substantial potency in clinical application and was presented as a new chemosensitizer agent in the treatment of esophageal cancer [108]. Using a low dose of paclitaxel with luteolin was shown to have synergistic effects on the regulation of esophageal cancer cell migration, proliferation, EMT, and apoptosis [108]. The proposed mechanisms to regulate cell migration and EMT processes include suppression of SIRT1 and activation of the mitochondrial apoptotic pathway through reactive oxygen species and the N-terminal kinase [108]. Coadministration of luteolin with the chemotherapeutic agent paclitaxel has been shown to increase cytotoxicity in previous research of estrogen-independent breast cancer cells (MDA-MB-231) [109]. The results showed that luteolin could inhibit stemless cancer and down-regulate antioxidant proteins. It could increase chemosensitivity via the Nrf2-mediated pathway [109]. Yang et al. proposed the combination of luteolin and paclitaxel as a novel strategy in breast cancer treatment. In MDA-MB-231 cells, luteolin and paclitaxel blocked STAT3 and increased the expression of caspase-8, caspase-3, and Fas to induce apoptosis [110]. In vivo data suggested that concurrent treatment with luteolin and paclitaxel significantly reduced tumor size and the total body weight of MDA-MB-231 cells from nude mice [110].

Based on previous study on a human tongue squamous cancer cells (SCC-4), the combined treatment of luteolin and paclitaxel improved the cytotoxicity of paclitaxel and continuous administration of this flavonoid could inhibit tumor growth inhibit in animal model [105].

## Morin (2′,3,4′,5,7-Pentahydroxyflavone)

In the scientific literature, morin has been presented as a component that has a flavonol structure and is one of the dietary sources available to humans; apple, tea, coffee, onion, mulberry, and almonds are just some fruits and vegetables that contain it, but there are many more [111]. Previous reports showed the usage of morin in the treatment and prevention of chronic diseases related to inflammation progression and oxidative stress, and it has been reported as a chemotherapeutic and chemopreventive agent [111]. Reduced oxidative stress, weakening of inflammatory mediators, downregulation of p-Akt and NF-κB expression downregulation, and activation of phase II enzymes were introduced as cancer prevention mechanisms. Apoptosis, ROS, cell cycle, MMPs, EMT, miRNAs, STAT3, PI3K)/Akt, MAPK, Hippo signaling pathways were reported as molecular targets of morin [111, 112]. According to a 2017 study, morin was suggested as a potential adjuvant agent of paclitaxel in prostate cancer by regulating the miR-155 / GATA3 axis. It has the potential to increase paclitaxel chemosensitivity in prostate cancer models conducted in vitro and in vivo [113].

## Myricetin (3, 5, 7, 3′, 4′, 5′-hexahydroxyflavonol)

Myricetin with a polyhydroxyflavonol structure was originally isolated from *Myrica rubra*. Evidence from the scientific literature points to the possibility of beneficial effects on health, including antioxidant, anti-inflammatory, antimicrobial, anti-obesity, anticancer, neuroprotective, and hepatoprotective properties of particular components [114]. Myricetin has been shown to have anticancer properties and may stop the growth, migration, and invasion of tumor cells. Furthermore, it causes cancer cells to commit suicide and alters cancer markers associated with the immune system. To fully realize its medicinal potential, further study is required [114]. According to a 2017 published study, myricetin could induce cytotoxicity and apoptosis and also inhibit the migratory capacity in human ovarian cancer cells. Furthermore, myricetin improved the paclitaxel chemotherapeutic ability of paclitaxel in human ovarian cancer cell lines by targeting multidrug resistance protein-1, in such a way that the expression of multidrug resistance protein-1 was considerably down-regulated compared to untreated cells and could be related to the increase in paclitaxel efficacy in ovarian cancer cells [115].

## Naringenin (4’,5,7-trihydroxy flavanone)

Flavanones such as naringenin are most often found in citrus fruits such as grapefruit and orange. Naringenin is a flavanone structure. Due to the lipophilic nature of naringenin, it is readily absorbed by enterocytes via the epithelium of the digestive tract through passive diffusion [116]. This phytoestrogen molecule has been linked to a variety of health benefits, including those related to the fight against cancer, inflammation, and prevention. Alterations in the ERK1 / 2 MAPK and PI3K/AKT signaling pathways have been shown in the past [117, 118]. According to the results of the studies by Lim et al. on prostate cancer cells, naringenin could induce apoptosis by regulating PI3K/AKT and suppressing ERK1 / 2, P38 and JNK. It could induce MMP loss and ROS generation for intrinsic apoptotic pathways in the PC-3 prostate cancer cell line, While ROS production occurred without change in MMP in LNCaP prostate carcinoma cell line, the results revealed that naringenin showed synergistic effects with paclitaxel and could improve the efficiency of paclitaxel to suppress cancer cell progression [117].

## Naringin (4’,5,7-trihydroxy flavanone-7-rhamnoglucoside)

Naringin with flavanone glycoside structure occurs in genus *Citrus* fruits, especially grapefruit [119]. Naringin is a naturally occurring molecule that plays a role in several molecular pathways, giving it a wide range of pharmacological and biological effects. Antioxidant, anti-inflammatory, bone regenerative and cancer preventing properties have been proven. Decades of study suggest that naringin might have a wide variety of practical uses [119, 120]. This bioflavonoid can influence several molecular pathways such as the PI3k, AKT, mTOR, AMPK, Nrf2, and iNOS signaling pathways [119]. They showed that apoptosis activation and a G1 cell cycle arrest handled the inhibitory effect of naringin on cell viability and that these effects were dose- and time-dependent. In DU145 cells, naringin increased BAX, BID, caspase 3, cytochrome c, p53, p21Cip1, and p27Kip1 mRNA levels while decreasing survivin and livin. These effects were shown in relation to the pathways that were evaluated [120]. Combining naringin with paclitaxel increased the cytotoxicity of paclitaxel in DU145, PC3, and LNCaP cell lines. Additionally, NF-κB p50 was downregulated and PTEN expression was up-regulated in DU145 cells after treatment with naringin or naringin with paclitaxel. In the end, they concluded that naringin served as a chemosensitizer and increased the cytotoxic potential of paclitaxel in prostate cancer cells [120].

## Nobiletin (3′,4′,5,6,7,8-Hexamethoxyflavone)

Nobiletin is a kind of polymethoxyflavone found in citrus fruit peels and has been associated with several health advantages. These include protection against free radical damage, inflammation, cancer, dementia, atherosclerosis, diabetes, and obesity [121–123]. Based on previous studies, a series of signaling pathways such as AMPK, PI3K/Akt, MEK/ERK, NF-B, Ca2+/CaMKII, TGF, HIF-1, could be involved in the emergence of biological effects [121, 123]. This natural flavonoid has been shown to inhibit the Nrf2/AKT/ERK pathways, which can increase paclitaxel concentrations in tumors and reverse paclitaxel resistance in a multidrug resistant xenograft model of cancer [124].

## Oroxylin A (5,7-dihydroxy-6-methoxyflavone)

*Scutellaria* baicalensis was the plant that led to the discovery of the flavonoid structure known as oroxylin A, which was found in the roots of the plant. *Scutellaria* baicalensis was the plant that led to the discovery of the flavonoid structure known as oroxylin A, which was found in the roots of the plant. Due to the broad spectrum of pharmacological effects, oroxylin A has attracted the attention of researchers around the world [125]. Strong anticancer activities were reported as the properties of this phytochemical, which was carried out through apoptosis induction, metestasis and invasion, reverse of multidrug resistance reversing, and suppression of angiogenesis suppression [125]. According to previous reports, oroxylin A acted as a P-glycoprotein-mediated cellular efflux inhibitor and affected the relative bioavailability and cytotoxicity of paclitaxel. Because of this, oroxylin A can help increase the cellular availability of P-glycoprotein substrates such as anticancer drugs [101, 126].

##  Quercetin (3,3′,4′,5,7- pentahydroxyflavone)

Quercetin is one of the most abundant and studied flavonol structures that can be found in different parts of herbs [127, 128]. It can show useful effects on the physical health of humans through mediating antioxidant processes, modulating effects on immune systems, and regulation of metabolic pathways, which are related to gene expression and modulation of signaling pathway activities [127]. The signaling pathways in various cancers were PI3K, AKT, mTOR, MAPK, ERK, JAK, STAT3, EGFR, AMPK, ERK1/2, S473AKT, Ras, ErbB2/ErbB3, JNK1/2 [127]. According to research presented in a paper published in 2005, quercetin could increase KB-V1 cells from human cervical carcinoma to paclitaxel and vinblastine, as well as decrease their relative resistance to these two anticancer agents in KB-V1 cells, which had a high plasma membrane P-glycoproteins and multidrug-resistant properties. After quercetin treatment, a significant increase in paclitaxel-induced cytotoxicity was observed [57]. It was introduced as a flavonoid structure with a latent inhibition of P-glycoprotein-mediated efflux [73]. Increased levels of the 61 cyclsteine-rich angiogenic inducer have been reported to increase proliferation, invasion, and resistance to apoptosis and paclitaxel in breast cancer cells [129]. Quercetin was found to be the most effective of the flavone structures tested against human gastric adenocarcinoma cells (AGS-cyr61) that had developed resistance to chemotherapeutic drugs such as paclitaxel due to overexpression of the cysteine-rich angiogenic inducer 61. This resulted in the viability of a significant reduction in AGS-cyr61 cells. This flavone structure suppressed colony formation, reversed multidrug resistance, triggered caspase-dependent apoptosis, hindered migration, and caused down-regulation of EMT-related proteins in AGS-cyr61 [129]. Lim et al. demonstrated that the combination use of quercetin and paclitaxel decreased the viability of choriocarcinoma JAR and JEG3 cells and the potency of this combination was greater than the potency of paclitaxel alone [130]. Furthermore, quercetin stabilized mast cell membranes, suppressed histamine release, and blocked PKC-dependent activation of transient receptor potential cation channel subfamily V member 1 to reduce paclitaxel-induced neuropathic pain in ‘in vitro’ and ‘in vivo’ tests [131]. In the other study, quercetin could attenuate the cell cycle activities of two co-administrated microtubule targeting drugs (nocodazole and taxol) in a short period, however, the combination of quercetin and taxol could reduce the clonogenicity and survival of cancer cells [132]. Plasma concentration, half-life, mean residence time, absolute bioavailability, and relative bioavailability of paclitaxel increased significantly after quercetin pretreatment prior to oral delivery in an animal model [133].

## Sciadopitysin

Sciadopitysin amentoflavone-type biflavonoid structure with interesting biological functions such as anticancer, antioxidant, osteoporosis treatment, diabetic osteopathy and neuroprotective effects [134]. Scadopitysin inhibited the P-glycoprotein in MDR1-MDCKII cells, decreasing taxol efflux and increasing cell toxicity [101]. The cell viability of MX-1 and MX-1/T cell lines was also reduced and its ability to increase taxol cytotoxicity was shown [101].

An extract containing paclitaxel (2.50%) and sciadopitysin (7.67%) produced a 60.85–93.91% inhibition rate of 600 mg/kg in xenograft models of human cancer that was named HDS-1. An anticancer effect is observed in nude A549-bearing mice that receive the paclitaxel-containing extract. HDS-1-derived flavonoids and lignoids, in addition to improving the rate at which paclitaxel is absorbed by enterocytes, significantly increase the level of cell death caused by BCL-2 [135].

Corroborating evidence that HDS-1 has anticancer action when taken orally comes from research on Taxus yunnanensis Cheng et L.K. Fu. These data demonstrate that HDS-1 acts as an endogenous bioenhancer and cytotoxicity enhancer for paclitaxel, increasing its oral bioavailability and anticancer effectiveness [136]. The use of HDS-1 at low doses showed greater sustainability and fewer side effects than when administered at higher doses, so adjusting its dosage may be necessary to achieve better results. Therefore, it is possible to use HDS-1 long-term as cancer treatment under appropriate dose adjustments. Research on the fundamentals of Chinese medicine places an emphasis on the complementary roles played by active and supportive ingredients [135].

## Silibinin

Silibinin, as a natural polyphenol with a flavonolignan structure, is an important and active ingredient in silymarin (a standardized mixture with a flavonolignan essence extracted from the seeds of Silybum marianum L. or milk thistle). Silibinin is a 1:1 combination of silybin A and B. In cultured cancer cells, milk thistle flavonolignan combinations have shown antiproliferative and antiangiogenic effects [137]. Silibinin increased oral bioavailability of paclitaxel by inhibiting the P-glycoprotein and the Cytochrome P450 3A4 subfamily in the small intestine and liver. Silibinin increased paclitaxel absorption [138]. Past preclinical research has shown the potent ability of silibinin to target the migratory and invasive properties of cancer cells. [139]. It could target signaling molecules involved in the regulation of EMT, protease activation, motility, adhesion, invasiveness, supportive modules of the tumor microenvironment, and inhibit metastasis to other distant organs [139]. A 2017 study on the MCF-7 cell line found that combining paclitaxel with silibinin improved treatment results [140]. In this study, the enhancement in early apoptosis occurred from 25.7% (paclitaxel alone) to 56.8% (silibinin and paclitaxel) and significant reduction in the antiapoptotic Bcl-2 gene with increasing levels of Bax, P53, BRCA1, and ATM mRNA [140]. Silibinin reduced invasion and migration in 786-O cells of renal cancer without cytotoxicity in a dose-dependent manner [141]. Furthermore, reductions in tumor weight and volume were observed by feeding silibinin in the animal model. Co-administration of silibinin and paclitaxel enhanced chemosensitivity of this chemotherapeutic drug [141].

Non-metastatic breast cancer receiving a regimen of doxorubicin / cyclophosphamide / paclitaxel treatment regimen used silymarin for the treatment of chemotherapy-induced hepatotoxicity in a randomized, triple-blind, placebo-controlled clinical trial investigation. Once silymarin therapy was continued for a full month, the findings showed a significant decrease in the severity of hepatotoxicity [142]. According to studies on the interactions of the flavonoid-P-glycoprotein substrate, paclitaxel had a modest interaction with biochanin A as a substrate of P-glycoprotein, as shown by a low area under the plasma concentration-time curve after oral and intravenous dosing [143].

## Sinensetin (3′,4′,5,6,7-pentamethoxy flavone)

Polymethoxylated flavonoid sinensetin was first isolated from Orthosiphon aristatus and later found in the fruits of many citrus species. Previous in vitro and in vivo research has revealed antioxidant, anti-inflammatory, anti-obesity, antimicrobial, anti-dementia, anti-angiogenesis, anticancer, and vasorelaxant properties [144]. Inhibition of the P-glycoprotein by sinensetin has been shown to increase paclitaxel cytotoxicity in MX-1 and MX-1/T (taxol-resistant cells), as reported in the study by Bai et al. [101].

## Tangeretin (5,6,7,8,4-pentamethoxyflavone)

The citrus fruit peel contains tangeretin, a non-toxic poly methoxylated flavone. Tangeretin’s advantages include antioxidation, inflammation, asthma, diabetes, neuroprotection, renoprotection, hepatoprotection, control of melanogenesis, immunomodulation, and tumor suppression [145]. TNF-α, iNOS, JNK, Nrf2, ERK, PI3K, Akt, and COX-2 are some of the signaling pathways affected by tangeretin [145]. Using direct suppression of ABCB1 transporter activity, tangeretin has been shown to make cancer cells more sensitive to chemotherapeutic drugs in a study published in 2016 [83]. Co-administration of tangeretin and paclitaxel stimulated apoptosis and arrested the G2 / M phase cell cycle [83]. Synergistic effects were observed between this methoxyflavone structure and paclitaxel in the treatment of human ovarian cancer cells resistant to paclitaxel (A2780 / T) and human NSCLC (A549/T) [83]. This flavonoid structure was also shown to increase paclitaxel cytotoxicity and reduce cell viability in MX-1 and MX-1/T cells in another investigation that evaluated the effects of tangeretin on paclitaxel-induced cytotoxicity [101].

##  TMF (7,3’,4’-trimethoxyflavone)

7,3’,4’-trimethoxyflavone is the other bioactive flavonoid structure with noticeable wound healing and cytotoxic effects [146]. According to the in vitro experiments by Jeong and Choi, TMF was used as a P-glycoprotein inhibitory agent to improve the bioavailability of paclitaxel in the human colon carcinoma cell line (Caco-2). It could improve paclitaxel cytotoxicity and transport (apical to basolateral). When this flavonoid structure was loaded onto the apical membrane of lung cancer cells, paclitaxel became more effective against SK-MES-1/PT4000 cells that overexpress the P-glycoprotein on the basolateral side [147].

##  Vadimezan (ASA404 or 5,6-dimethylxanthenone-4-acetic acid)

Vadimezan is known as the analogue of flavone-8-acetic acid with tumor vascular disrupting and tumor hemorrhagic necrosis actions. Decreasing tumor blood flow, increased vascular permeability, and endothelial apoptosis were reported as the results of ASA404 administration in murine tumors. It could induce an increase in the concentration of TNF and some other cytokines in tumor tissue [148]. Vascular effects in humans were reported in a phase I clinical trials study. In addition, it could reveal significant cytotoxic activity in co-administration with paclitaxel and carbopolatin in a phase II trial [148]. Negative effects were reduced when ASA404, paclitaxel, and carbopolatin were administered together to Japanese patients with NSCLC, according to the results of phase I clinical trials released in 2011 [149]. In the other phase II clinical study, in which 108 patients with squamous and non-squamous NSCLC were included, the addition of ASA404 to standard treatment (paclitaxel and carbopolatin) did not result in increased toxicity and no serious side effects related to bleeding, pulmonary hemorrhage, or hemoptysis were reported [150]. Furthermore, the combination of ASA404 with standard treatment has the potential to improve survival rates in both squamous and nonsquamous populations [150].

##  Others

Aurones are a type of flavonoid compound that has a 2-benzylidene-coumaran-3-one as their fundamental structure. These structures may be connected to hydroxyl or O-substituted hydroxyl groups that are in the aromatic ring. They are to be credited for the brilliant yellow hue that may be seen in some varieties of beautiful flowers [151]. Four aurone structures (4,6-dimethoxyauronie derivatives and 4-hydroxy-4-methoxyaurone) modulated paclitaxel transport in resistant breast cancer cells better than 13 flavonoid structures [35].

The continuation of reviews of the past literature showed several studies on the role of plant extracts on the effects of paclitaxel, and the results were categorized in Table 2:

**Table 2:** Plant extracts and paclitaxel Co-administration results

Others	Plant name	Study design	Flavonoid content	Plant dosage	Paclitaxel dosage	Duration of study	Mechanism of action	References
1	*Camellia sinensis*: 2 fractions of (FLG: flavonol glycoside, FLA: flavonol aglycone)	In vitro: DLD-1 and E0771 cells	FLG: 132.76 mg catechin equivalents/g dry weight FLA: 174.67 mg catechin equivalents/g dry weight	10 and 100 µg/mL of FLG and FLA	10 nM	24 h	Synergistic anti-cancer effects in the treatment with FLG and FLA combined with paclitaxel Inhibited synergistically growth of cells	[152]
2	*Kaempferia parviflora* (ethanol extract)	In vitro: HL 60 cells	-	40 µg/mL	10–50 µM	24, 48 and 72 h	Inhibited cell growth and reduced cell viability Apoptotic cell death and loss in mitochondrial transmembrane potential and activation of caspase 3. Improved apoptosis through synergistic effect	[153]
3	*Morus alba* (water extract)	In vitro: TSGH 8301 In vivo: Four-week-old BALB/c male nude mice	-	In vitro: 0–1500 µg/ml In vivo: 4 mg/kg/ day for 10 weeks	In vitro: 3 nM In vitro: 147 nM, once a week for 9 weeks	In vitro:24 and 48 h In vitro: 10 week	Bladder carcinoma cell death by the cell cycle arresting at the mitotic phase. Induced mitotic catastrophe and impaired the early endosome generation Induced the PTEN activation and expression and also inhibited early endosome formation Retarded tumor growth in a human bladder carcinoma model	[154]
4	*Orysa sativa* ,Doisaket, Nan, and Payao cultivars (methanol and dichloromethane extract)	In vitro: HepG2, LNCaP, NIH3T3	Anthocyanin content of methanol extract of Payao cultivar: **5.80 mg/g**	In vitro: 0-200 µg/ml, The IC20 value of methanol extract of methanolic Payao extract on HepG2: 175.95 µg/ml	In vitro: The IC20 value of paclitaxel for 24 and 48 h were 0.105 and 7.8 µM for HepG2	In vitro:24 and 48 h	Induced cytotoxicity is not synergistic or antagonistic, but additive	[156]
5	*Polygonum minus* (methanol extract)	In vivo: male Sprague-Dawley rats (weight 180 ± 20 g)	-	In vivo: 200 and 400 mg/kg; orally	In vivo: 2 mg/kg/10 days; IP	In vivo: 10 days	Administration of the *P. minus* methanolic extract attenuated paclitaxel-induced mechanical hypersensation in a dose-dependent manner in pinprick test. Administration of the *P. minus* methanolic extract reduced paclitaxel-induced thermal hyper-sensation in a dose-dependent manner in tail-flick test and plantar test	[157]
6	Ethanol extracts of *Sophora flavescens* Aiton roots (KS-Fs	In vitro: H460, Caco-2 and Eca-109 cells In vivo: H460 and Eca-109 xenografted tumor models (female, 4–6 weeks old, 20–22 g)	kurarinone (29%), 2′-methoxy-kurarinone (5%), sophoraflavanone G (2%) and other minor flavonoids species	In vitro: 20 µg/mL In vivo: Flavonoid fraction: 200 mg/kg/day and kurarinone (100 mg/kg/day)	In vitro: 10 ng/mL In vivo: 5 and 10 mg/kg/day.	In vitro: 48 h In vivo: 21 days	Flavonoid fraction and kurarinone were able to improve the taxol effects on tumor cell line proliferation in vitro/in vivo	[158]

Bold indicates total anthocyanins of Payao-purple rice extracts (PYO-PRE) were 5.80 mg/g

1. ***Camellia sinensis*** **(Family: Theaceae)**

In a previous study on the antioxidant, anti-inflammatory and anticancer effects of two fractions of green tea, flavonol glycoside (containing 16 derivatives such as two apigenin glucosides: apigenin-6-C-glucosyl-8-C-arabinoside and apigenin-6-C-glucoside) and flavonol aglycone, in cell lines of colon adenoma and breast cancer, the results showed synergistic anticancer effects. Furthermore, the growth of colon adenoma and breast cancer cells was synergistically inhibited [152]. This study reported that flavonol glycoside and flavonol aglycone considerably decreased inflammation-related expression of the mRNA gene related to inflammation in murine RAW 264.7 macrophages [152].

2. ***Kaempferia parviflora*** **(Family: Zingiberaceae)**

In a 2008 published study, *Kaempferia parviflora* (Thai ginseng, or black ginger) with a high content of flavonoids was used to evaluate apoptosis in HL-60 cells. The results showed synergistic apoptosis in paclitaxel and ethanolic extract of the rhizomes of *K. parviflora* rhizomes co-treatment in human myeloid leukemia cells (HL 60) [153].

3. ***Morus alba*** **(Family: Moraceae)**

Human bladder cancer cells were tested for a synergistic impact between mulberry fruit water extract (a primary source of phenols and flavonoids) and paclitaxel by the Chen et al. They found that the combination of mulberry extract with paclitaxel increased the cytotoxic action of paclitaxel, leading to a more severe arrest of G2 / M, mitotic catastrophe, and subsequent apoptosis [154]. Observing differences in Aurora A and PLK1 expression among combined treatment with mulberry paclitaxel and paclitaxel alone proposed the appearance of a defect in cytokinesis early steps of cytokinesis [154]. Mulberry-paclitaxel reduced immunofluorescence staining of the early endosome antigen 1 and improved PTEN expression, representing the inhibition of endosome recycling endosome formation that was essential for cytokinesis [154]. Mulberry-paclitaxel treatment in the in vivo study of the TSGH 8301 xenograft model retarded tumor growth by activating PTEN and Caspase 3 activation [154].

4. ***Oryza sativa*** **(family: Poaceae)**

Purple rice or Oryza *sativa* is known as one of the most complete and nutritious types of rice, which is rich in antioxidant constituents and, for this reason, is useful in the control of many oxidative stresses, such as cancer [155, 156]. According to a previous study on the effects of purple rice extracts (*Oryza. sativa* var. indica) on paclitaxel-induced cytotoxicity in cancer cells, the observed results were neither synergistic nor antagonistic, but additive [156]. The methanol extract of the Payao cultivar with an acceptable level of anthocyanin content was the most potent cytotoxic extract in HepG2 cells [156].

5. ***Polygonum minus*** **(Family: Polygonaceae)**

The other study showed that *polygonum minus* methanolic extract could improve paclitaxel- and scopolamine -induced neuropathic pain and cognitive dysfunction in animal model evaluation. The neuroprotective activities of the extract could be related to its significant antioxidant functions, inhibition of lipid peroxidation, regulation of anti-inflammatory and cholinergic neurotransmitters [157].

6. ***Sophora flavescens*** **(Family: Leguminosae)**

Kushen or *Sophora flavescens* is a Chinese herbal medicine with important anti-inflammatory and anticancer properties [158]. The flavonoid fraction of dried roots of S. flavescens was found to be composed of kurarinone, 2′-methoxy-kurarinone, sophoraflavanone G, and other minor flavonoid compounds, according to a study by Sun et al. When tested with taxol, the flavonoid fraction and kurarinone were shown to have synergistic effects on proliferation and tumor development [158].

Enhanced clinical efficacy may be possible through further investigation of the immunological foundation and other potential mechanisms of action, as well as the development of new dosage regimens and/or administration methods.

**Fig. 2:**
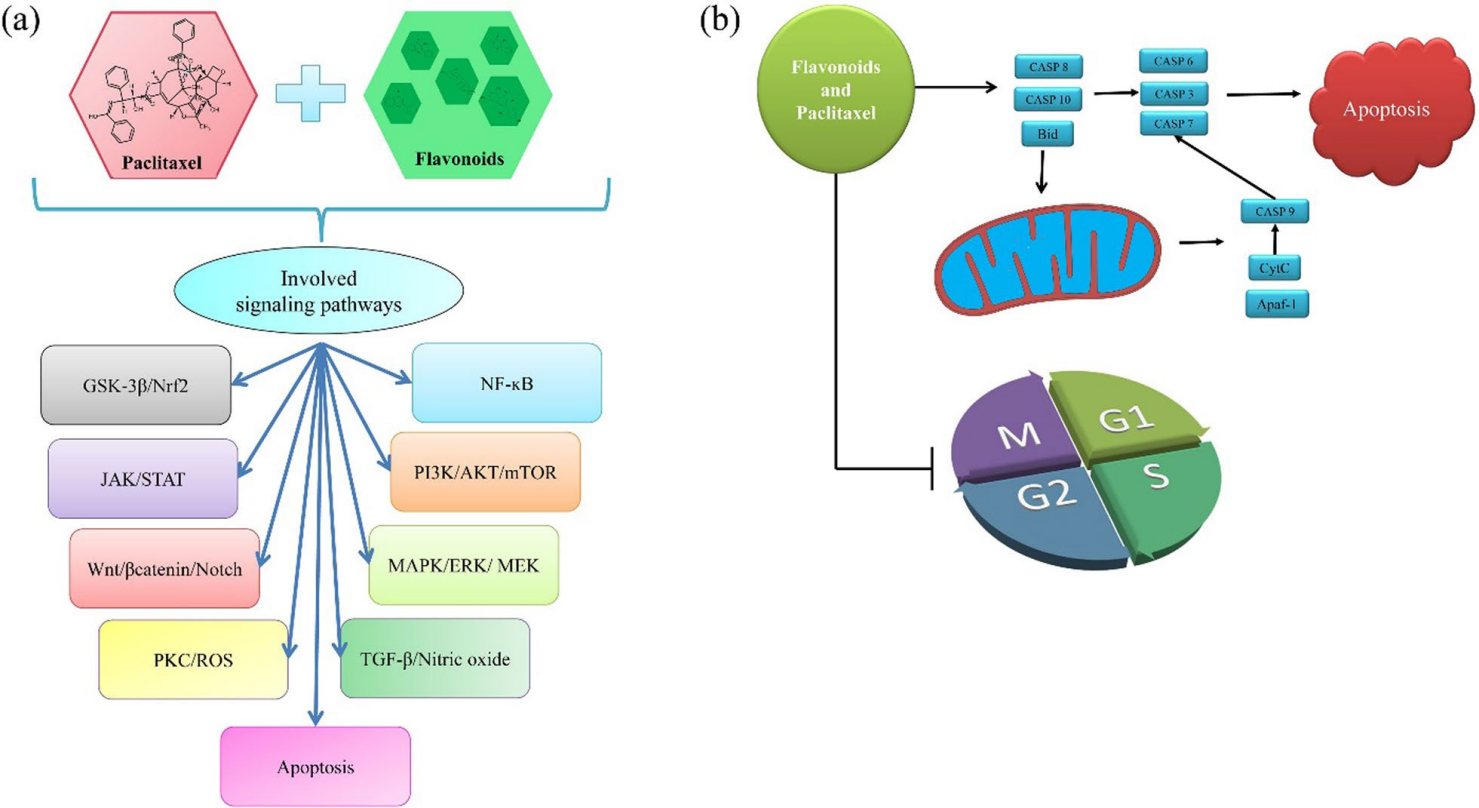
Summary of mechanism of action for synergistic effects of Paclitaxel and Flavonoids. **a** The involved signaling pathways are demonstrated leading to improved anti-cancer effects, **b** Activation of apoptosis and inhibition of cellular functions such as proliferation through cell cycle arrest

Finally, Fig. 2 summarizes the involved mechanism schematically when paclitaxel and flavonoids are administered simultaneously. Despite this, drugs are tested in clinical trials on a variety of genetically diverse patient populations to improve their likelihood of responding to treatment and reduce the likelihood of acquiring resistance. Combinatory chemotherapy drugs that include paclitaxel and different types of flavones must also be based on preclinical evidence from human trials. In the future, it could be useful as a therapeutic drug if additional research is done on its tumor growth-inhibition properties.

## Conclusion

In this work, we investigate the potential benefits of combining the chemotherapeutic agent paclitaxel with beneficial flavonoid chemicals to combat various issues, such as drug resistance and side effects that arise during cancer treatment. The study findings showed synergistic benefits, decreased toxicity, decreased drug resistance, etc. This research showed that flavonoids have the potential to act as an adjuvant therapy for all malignancies.

## Data Availability

Not applicable.
